# Lennox gastaut syndrome, review of the literature and a case report

**DOI:** 10.1186/1746-160X-4-9

**Published:** 2008-06-09

**Authors:** Tareq Abu Saleh, Lawrence Stephen

**Affiliations:** 1 Department of Oral Medicine and Periodontics, University of the Western Cape, Cape Town, South Africa; 2 Future Dental and Implant Center, Jordan Hospital, Amman, Jordan

## Abstract

**Background:**

Lennox-Gastaut syndrome (LGS) is a severe form of childhood epilepsy that is defined by generalized multiple type seizures, slowness of intellectual growth, and a specific EEG disturbance. Children affected might previously have infantile spasms or underlying brain disorder but etiology can be idiopathic. In South Africa, the incidence of secondary epilepsy is higher than what is found in developed countries resulting in higher incidence of the disease. LGS seizures are often treatment resistant and the long term prognosis is poor.

**Report:**

A twenty six year old female, presented with anterior open bite, macroglossia, supragingival as well as subgingival calculus. The gingiva was red, swollen and friable and there was generalized bleeding and localized suppuration. The patient had gingival recession. After periodontal therapy a remarkable improvement in oral health status was noted.

**Conclusion:**

The clinical findings in LGS included facial deformities, periodontitis and gingival swellings. Interdisciplinary treatment of these patients is fundamental and oral attention is of outstanding importance. Non-surgical periodontal therapy was effective in controlling periodontal disease in the reported case, but prevention of periodontal and dental diseases is preferable for this high-risk group of patients.

## Background and literature review

Lennox-Gastaut syndrome (LGS) is one of the catastrophic childhood epilepsies. It is defined by a triad of symptoms:

- Multiple types of generalized seizures, which are difficult to control.

- Slowness of intellectual growth, often accompanied by mental retardation and behavioral problems.

- A specific electroencephalogram (EEG) pattern called a slow spike-and-wave pattern (< 2.5 Hz), which is present when the child is awake [1-4].

Seizures most often present between the second and sixth year of life, however; they can start a little earlier or later. They rarely start after the age of eight [2,3].

William Lennox described the clinical features of the syndrome in 1930s, and then Lennox and Davis reported the symptomatic triad of the syndrome. Later, Gastaut expanded on the original observations of Lennox and Davis [5,6].

The diagnostic criteria of the syndrome have been refined by researchers, but the basic characteristics of the syndrome remained unchanged since Lennox and Davis [7-11].

Distinguishing LGS syndrome from other epilepsy syndromes has been challenging as it is characterized by plethora of underlying causes, multiple types of seizures, and cognitive impairment. Seizures are classified according to the International League Against Epilepsy (ILAE) classification, and specific epilepsy syndromes of childhood are recorded when their essential diagnostic elements are fulfilled. It is now agreed that a number of individual syndromes, including LGS, form a spectrum of childhood epilepsies, each with differentiating criteria [12-14].

## Prevalence

The Lennox-Gastaut syndrome is uncommon but it is very serious. The mortality rate ranges from 3% to 7% [2,5].

Often the prevalence of LGS is described as a percentage of childhood epilepsy. The reported prevalence of LGS varies between studies but it is in the range of 3–10% of childhood epilepsy, and is more common in males than females [1-3].

Epilepsy is relatively common, but its prevalence varies widely affecting approximately 3.4/1000 of the population in Japan, 6/1000 in the United States, and 11.2/1000 in Mexico [15,16].

In South Africa, the prevalence of epilepsy (all ages) has been reported as 10 per 1,000 [17]. In rural South African children aged 2–9 years the prevalence of epilepsy was 0.73%. Out of the affected, 57.1% didn't receive medication, and 71.4% had developmental disability. These figures are slightly higher than those derived from other sub-Saharan African countries [15].

As LGS constitutes a fraction of childhood epilepsy it is overall rare. However, it is clinically prominent due to the frequent and difficult to treat seizures that persist into adulthood, as well as to the need for continuous medical attention [1].

Epidemiologic community based studies in the so-called developed countries revealed that the annual incidence of LGS in childhood was approximately 2 per 100,000 children, and the prevalence of LGS was 0.1–0.28 per 1000 in Europe [4]. Studies demonstrated that the figures of LGS are relatively consistent across the developed populations. For example, in Atlanta, USA, LGS accounts for 4% of patients with childhood epilepsy, with a reported incidence of 0.26 per 1000 live births [6]. The true incidence in different populations is not known, due to the disparity between parameters used to define the disease, as well as to the variability of the predisposing factors to the disease among different populations.

## Etiology

Etiology of LGS is variable; LGS does not usually run in families but genetic factors may play a role in the etiology [2,7]. In 20–50% of cases the child has previously had infantile spasms with underlying brain disorder (also known as symptomatic West syndrome) [3,5,7,18,19].

For about one third of the affected children a known cause cannot be identified. These cases are referred to as cryptogenic Lennox-Gastaut syndrome [2,3].

On the other side, many of the children who develop Lennox-Gastaut syndrome had a pre-existing brain disorder or injury. Causes identified include tuberous sclerosis, congenital infections, hereditary metabolic diseases, brain malformation, and brain damage (due to birth asphyxia or other birth injuries, encephalitis, meningitis or head injuries) [2,3].

These patients tend to have worse prognosis than those of cryptogenic etiology [2,3]. It was noted, however, that these potential causative factors are different in different populations, and that in developing countries LGS was mainly secondary to trauma and infections [17].

In developing countries, there is a high incidence of preterm and abnormal births. During infancy and early childhood, meningitis, tuberculosis, neurocysticercosis, and head trauma are common. This situation is expected to increase the prevalence of secondary (symptomatic) epilepsy and that of intellectual handicap in the child population [17].

In a study of childhood recurrent seizures in Red Cross memorial hospital in Cape Town, South Africa, 11% suffered from menigities or encephalitis as a precipitating factor and 55% were intellectually handicapped [20]. Most patients who had both grand mal and myoclonic seizures suffered from LGS [20].

In another study in Cape Town, It was found that 43% of childhood epilepsy was symptomatic (secondary) [17]. This figure is even higher than other figures found in studies conducted predominantly in tropical African countries [17].

Perinatal hypoxia, meningitis, granulomata (cysticercosis and tuberculosis), and trauma have high prevalence among the poor of the Western Cape, and it is expected that these conditions serve as precipitating factors of secondary epilepsy [17]. On the other hand diagnostic facilities are better in Cape Town than in the rest of the studied African cities, which is expected to reduce the number of overlooked cases, resulting in higher figures.

## Clinical features

Typically, daily multiple seizure types that occur in LGS are of wide range, more than that of any other epileptic syndrome. The major kinds of seizures that usually occur in LGS are the tonic seizures, which are often nocturnal [3], the atonic seizures (involuntary losses of muscle tone that cause drop attacks) or atypical absences (child goes blank lasting up to a minute) [3]. Additionally, about 60% of children may have prolonged or repeated seizures very close together. This is called status epilepticus and is an emergency [5]. Some children also have other types of seizures, such as myoclonic, partial or tonic-clonic seizures [1,3].

Most children with the Lennox-Gastaut syndrome have a degree of intellectual impairment and learning disability that ranges from mild to severe. Behavioral problems and depression are also common, which can be attributed to the brain injury, the frequent seizures, the lack of normal social stimulation or as side effects of AntiEpilepticDrugs (AEDs) [5,21]. Children with Lennox-Gastaut syndrome are also more likely to have cerebral palsy, progressive decline in IQ and progressive gait disturbances [5].

Development of the child is frequently retarded at the onset of disease, depending on the etiopathogenesis of the underlying brain disease [14].

The syndrome is also characterized by an interictal (between-seizures) EEG disturbance called slow spike-wave pattern (< 2.5 Hz), often accompanied by a burst of fast rhythms of 10 to 12 Hz at night [1-3].

As a result of all these abnormalities, the child may look irritable, tired or bored. Many children fail to cope with school and need institutional care. The child's development is rarely normal, and often there is delayed development or other forms of epilepsy. The seizures can cause sudden falls and/or loss of balance, and patients are advised to wear a helmet to prevent head, face and teeth injuries.

The effects of LGS on the child and his family require a team of health care professionals to provide the best seizure control, the highest level of function with the least side effects, and the maximum quality of life possible for the child and to direct families to appropriate community resources. These children and their families need the combined support of services from healthcare workers, caregivers, friends, school and social workers as well as psychologists.

## Treatment

LGS seizures are often treatment resistant. Basic treatment is pharmacological and some children may need more than one mode of treatment.

Pharmacological treatment consists mainly of one or multiple antiepileptic drugs (AED's). No single treatment regimen could be considered superior to the others, and management depends on the response of the patients. Medical treatment usually starts with valproates (valproic acid, sodium valproate and valproate semisodium) followed by adjunctive therapy with either lamotrigine or topiramate [5,21].

Controlled clinical trials demonstrated that felbamate was beneficial in patients with the LGS. However, since felbamate was associated with an increased risk of aplastic anemia and hepatotoxicity, it is used with extreme caution and requires regular monitoring of complete blood count and liver enzymes. It is reserved for children who are refractory to other therapies [22].

Benzodiazepines, (specifically clonazepam, nitrazepam, and clobazam) and Phenobarbiturates, are recommended as third-line choices in cases difficult to control or if standard treatment is intolerable [5,21].

### Surgical options for treatment include

-Vagus nerve stimulation: The procedure involves implantation of a battery-operated device with connecting wires to the left vagus nerve, which is programmed to deliver a current at variable frequencies, pulse widths, and times [23].

-Corpus callosotomy: an operation to cut the corpus callosum (the large bundle of nerve fibres connecting the two cerebral hemispheres) may be considered in the treatment of LGS [23].

Electrical stimulation of the centromedian thalamic nucleus (ESCM) was reported as being efficient in the control of generalized seizures and improvement of quality of life of the patients. The method involves stereotactic surgical implantation of electrodes to the centromedian nuclei of the thalamus. Electrodes are stimulated within set parameters. Patients are scheduled for follow-up visits for assessment of seizures and neurophysiologic tests. Main disadvantage was skin erosions that could not be controlled by plastic surgery procedures [24].

Ketogenic diet is a reportedly effective option for treatment. A ketogenic diet is a high fat, low protein, low carbohydrates diet that induces ketosis, a state in which there is an excessive amount of ketones in the body. It is very effective and is becoming increasingly popular for treatment of LGS [2].

## Prognosis

The long term outcome is poor in terms of seizure control and intellectual development. Catastrophic disorders such as LGS are associated with morbidity and mortality [1]. Eighty percent of patients with Lennox-Gastaut syndrome continue to have seizures throughout childhood and into their adult life. The mortality rate ranges from 3 to 7% [2].

To the best of our knowledge this is the first report that documents the oral clinical and radiographic findings as well as the response to treatment in a patient with LGS.

## Case report

A twenty six year old female with longstanding cryptogenic Lennox-Gastaux syndrome was referred to the department of Oral Medicine and Periodontics, University of the Western Cape, Cape Town, South Africa, complaining of swollen bleeding gums.

The patient was a "longstanding Gastaux-Lennox Syndrome case" with occasional generalized seizures, well controlled on Sodium Valproate, Phenobarbiturates and Lamotrigine. The patient did not experience any seizures in the last month before presentation. She was also diabetic (type II), well controlled on oral Hydralazine and Glucophage, and hypertensive controlled on: Atenolol and Adalat. This medical condition influenced the patient's abilities and consequently she was dependent on her family for daily living. Patient had fair intellectual activity.

### Extraoral examination

Patient had course facies, with bimaxillary protrusion, and her lips were incompetent at rest.

### Intraoral examination

**Figure 1:**
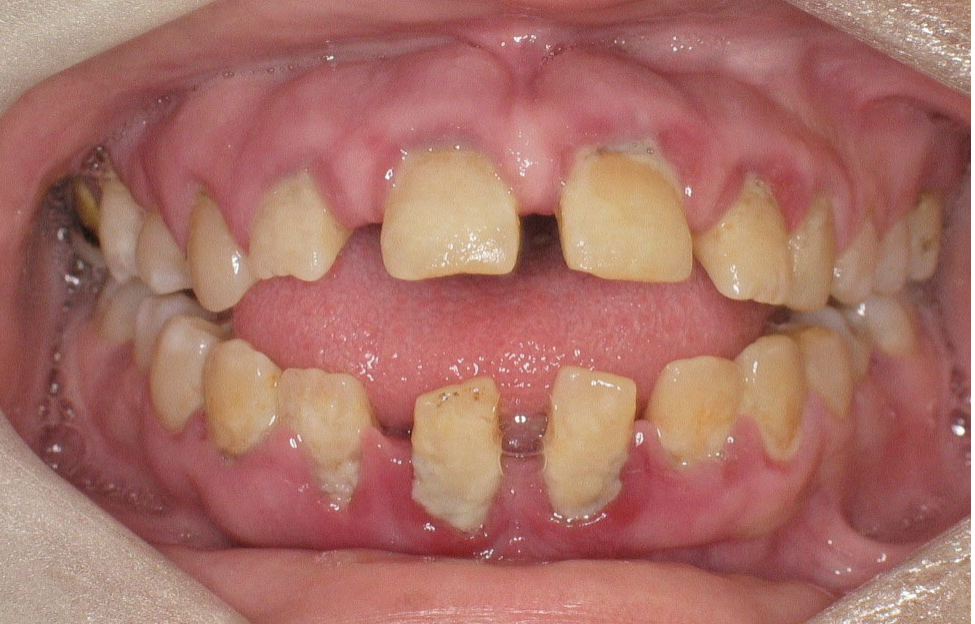
Anterior open bite and spacing is evident.

The patient had anterior open bite (Figure 1) and macroglossia which made it difficult to perform proper oral hygiene methods, especially posteriorly, and oral hygiene status was poor. All teeth were present and the patient had no restorations. Multiple teeth were carious (smooth surface caries in posterior teeth and pits caries in anterior teeth).

**Figure 2:**
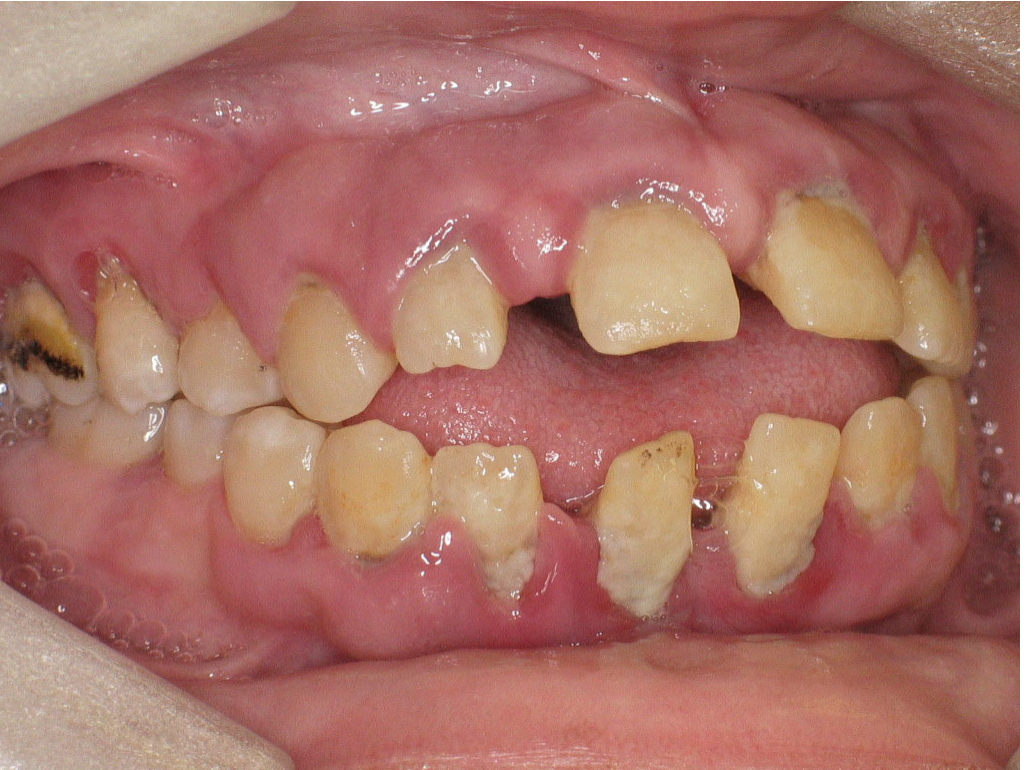
Inflamed gingiva, and plaque deposits.

There were large amounts of supragingival as well as subgingival calculus (Figure 2). The patient reported dental and periodontal sensitivity at the anterior teeth region.

**Figure 3:**
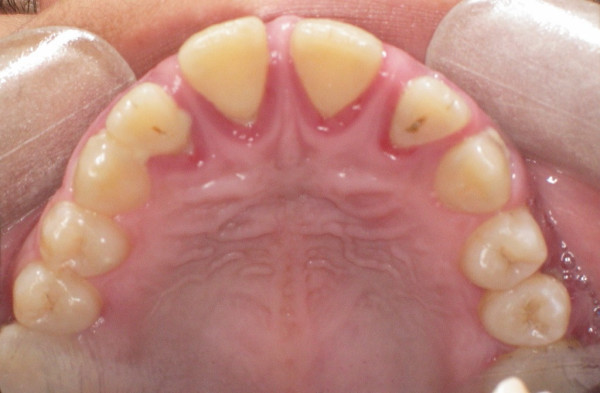
Marginal gingivitis and recession palataly.

The gingiva was red, swollen and friable and there was generalized bleeding and localized suppuration. The patient had gingival recession labially and lingually/palatally, especially anteriorly (Figure 3). The whole-mouth mean probing pocket depth was 4.7 mm and 30% of the periodontal pockets were deeper than 5 mm causing furcation involvement in some posterior teeth. Teeth # 11, 21, 31, 32, 41 and 42 (FDI) were mobile (grade two and three).

### Radiographic examination

**Figure 4:**
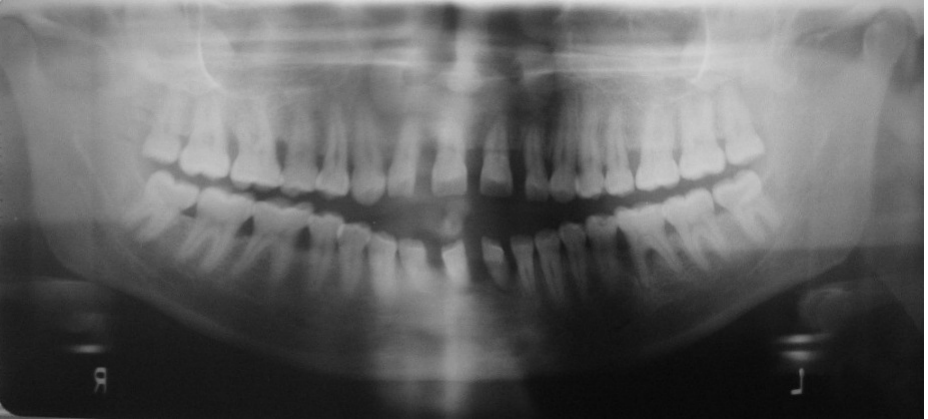
Orthopantomograph showed severe horizontal alveolar bone loss anteriorly, and vertical bone loss around 31 and 36.

An orthopantomograph showed severe horizontal alveolar bone loss, especially anteriorly, and vertical bone loss around 31 and 36. Spacing of teeth was evident anteriorly. Multiple proximal radiolucencies were present on posterior teeth (Figure 4).

Considering the oral clinical and radiological findings the recorded periodontal diagnosis was Generalized Moderate to Severe Chronic Periodontitis associated with systemic disease.

The proposed treatment plan was:

1) Comprehensive oral hygiene instructions and education to the patient as well as her family.

2) Extraction of teeth of poor prognosis when general health conditions allow, and provision of immediate dentures.

3) Subgingival scaling using ultrasonic applicators and hand instruments under local anaesthesia, and the use of 0.2% Chlorehexidine gluconate mouthwash.

4) Root planing of the four quadrants under local anaesthesia, with special care to the exposed furcations, and subgingival irrigation with 1% chlorhexidine solution.

5) Elective gingivoplasty/pocket reduction surgery for swollen segments with persistent deep pockets.

6) Vitality testing for teeth with questionable pulp vitality, and restoration of the carious lesions with filling materials and the edentulous areas by fixed porcelain fused to metal bridge.

7) Supportive periodontal treatment, with a three month recall period.

The patient received professional scaling and root planing distributed over multiple visits, and oral hygiene practices were emphasized. Three months following the treatment a remarkable improvement in oral health status was noted, viewed by absence of gingival inflammation, reduction of probing pocket depths negating the need for gingivoplasty or pocket reduction surgery, as well as absence of calculus or plaque deposits. Teeth # 31, 32, 41, 42 were extracted and an immediate denture was provided, and the patient was enrolled in supportive periodontal treatment phase consisting of professional tooth cleaning and motivation of oral hygiene every three months.

## Discussion

Lennox-Gastaut syndrome (LGS) is a catastrophic epileptic encephalopathy that consists of multiple types of generalized seizures which often vary in their frequency over a period of time. The syndrome is difficult to treat and many people receive multiple drugs (polypharmacy) without achieving satisfactory seizure control [23].

The long-term prognosis is poor; although the epilepsy often improves with time, complete control of seizures is rare and with time the mental and behavioural disorders tend to worsen [23].

In managing patients with LGS the type of seizures, the level of seizure control, the intellectual activity of the patient and the possible drug reactions should all be considered [25].

In a study to determine oral health status and treatment needs for caries and periodontal lesions, phenytoin induced gingival hyperplasia and fractures of anterior teeth, in 12–13 year old epileptic children in South Africa, it was found that these children were a high risk group and should be regarded as a priority group for dental treatment. It was recommended to shift oral health programs from treatment into primary preventive care in these (high risk) groups [26].

The reported case is typical for LGS patients who may present for dental/oral health care. The patient experienced LGS and she was controlled on three drugs (Polypharmacy) including Phenobarbiturates. A report from South Africa indicated that more than half of the children with recurrent seizures in Red Cross memorial hospital in Cape Town had received Phenobarbiturates at some stage, and although this drug is not favored in developed countries because of its adverse effects on behavior and the higher success rate with carbamazepine, phenytoin, or valproic acid, it is still widely used in South Africa because it is cheap and effective [20]. The patient was still experiencing occasional generalized seizures. The choice of medication is usually related to individual tolerance and efficacy, but it is important to note that all AEDs produce significant -though variable- side effects, including blood dyscrasias, anemia, and alteration of hepatic function, which can deteriorate blood clotting and complicate general anesthesia [27]. No such side effects were detected in the patient.

Thickening of the heel pad and of the calvarium, coarse facies, congenital anomalies of the face, microstomia and macroglossia have also been reported. Consistent with the literature some findings such as macroglossia and coarse facies were noted in the patient but the exact association with the syndrome was not disclosed [27,28].

Seizures may occur several times everyday, and patients suffering drop attacks might have fractures in the head and face region. Severe cases may interfere with school and social activities exacerbated by medications especially phenobarbiturates.

No history of trauma to the face or teeth was recorded but the patient had poor communication skills and she was dependant on her family.

Patients on AED's are subject to gingival overgrowth, mostly associated with using phenytoin. About half of the patients placed on phenytoin will show evidence of gingival enlargement, usually within 2 to 18 months after starting the medication [25]. The etiology is still unknown, but there appears to be an increase in the number of fibroblasts in the connective tissues [27]. Valproate is also associated with gingival hyperplasia but less than phenytoin [29]. Polypharmacy itself may predispose the patient to gingival enlargement [30].

In literature, it was noted that there is a correlation between poor oral hygiene and the amount of tissue enlargement [30]. The reported patient had gingival enlargement which could be a side effect of AED's aggravated by poor oral hygiene, but also it could be complicated by diabetes or hormonal influences.

Management starts by professional cleaning measures that can reduce the size of the swollen edematous tissue, and for persisting swollen tissue gingivoplasty/gingivectomy is performed preferably by radiosurgery to reduce bleeding at time of operation. The patient was treated only by professional scaling and root planing which reduced swellings and probing depths. Mobile teeth were extracted. Ultimately surgery wasn't necessary.

The patient experienced multiple carious lesions and large amounts of plaque deposits that could be attributed in part to the negligence of oral hygiene or due to her reluctance to brush because of the bleeding gingivae. Macroglossia – which is in turn could be reactive to continuous use of AED's during years of development or secondary to diabetes mellitus – might complicate oral hygiene methods especially posteriorly.

Patients placed on AED's therapy should be referred to a dentist/periodontist for plaque control instructions and education, professional scaling or gingival curettage and regular follow-up visits.

To restore partial edentulism it is generally preferable to place fixed prosthesis rather than removable appliance because of the possibility for removable appliances to become dislodged during seizure attacks. It is also advised to use metallic prosthesis as it is less liable to fracture.

Costs of treatment also should be considered if prostheses are expected to be replaced because of the repetitive fractures. Ultimately, for every case a treatment plan should be designed individually [25].

Fillings can be done safely, provided that the dentist is aware of the level of seizure control and intellectual activity of the patient as well as the difficulty to apply local and general anesthesia. Coordination with the physician/neurologist is important in this regard.

## Conclusion

The clinical findings in LGS could be associated with the syndrome itself or with the effects of pharmacological treatment, and it might include facial deformities, periodontitis and gingival swellings. Interdisciplinary treatment of these patients is fundamental and oral attention is of outstanding importance.

Non-surgical periodontal therapy was effective in controlling periodontal disease in the reported case, but prevention of periodontal and dental diseases is preferable for this high-risk group of patients.

## Competing interests

The authors declare that they have no competing interests.

## Authors' contributions

TA and LS conceived the study, TA reviewed the literature and LS drafted the manuscript.
